# Vascular endothelial growth factor-A level in human breast milk after intravitreal injection of ranibizumab: a case report

**DOI:** 10.1186/s13006-022-00463-y

**Published:** 2022-03-31

**Authors:** Ying Huang, Rong Zhou, Zuhua Sun, Yihan Zheng, Bing Lin

**Affiliations:** Eye Hospital and School of Ophthalmology and Optometry, Wenzhou Medical University, Wenzhou, Zhejiang, China

**Keywords:** Vascular endothelial growth factor-a, Human breast milk, Intravitreal injection, Ranibizumab

## Abstract

**Background:**

Ranibizumab is one of intravitreal anti-vascular endothelial growth factor agents. It is applied in the treatments of choroidal neovascularization, age-related macular degeneration, diabetic macular edema, and macular edema secondary to retinal vein occlusion**.** Preliminary evidence suggests that intravitreal ranibizumab may enter the plasma and human breast milk in very low-level concentration. As a precaution, breastfeeding is not recommended during the treatment of intravitreal injection of ranibizumab. There are limited data regarding the change of anti-vascular endothelial growth factor concentration in human breast milk after intravitreal injection of ranibizumab, especially in the first 24 h after injection. The purpose of this report is to analyse the concentration change of vascular endothelial growth factor-A in human breast milk with time, in the short term after intravitreal injection of ranibizumab.

**Case presentation:**

In June 2018, a 30-year-old patient breastfeeding a six-month-old baby was diagnosed with choroidal neovascularization of left eye in Eye Hospital of Wenzhou Medical University. She received four administrations of 0.5 mg intravitreal injection of ranibizumab of the left eye, and breast milk was collected just before the injection, and 1–3, 6, 12, 24, 48, and 72 h after intravitreal injection, and assessed for vascular endothelial growth factor-A concentration. The change in vascular endothelial growth factor-A concentration in human breast milk showed the same trend after each injection, decreasing significantly within 6–12 h (about 20–30% lower), and increasing to pre-injection level by 24 h after injection.

**Conclusions:**

The concentration of vascular endothelial growth factor-A in human breast milk of a mother who continues lactating dropped initially and rose to pre-injection level about 24 h after intravitreal injection of ranibizumab. The data may offer more information to evaluate the impact of anti-vascular endothelial growth factor agent intravitreal injection of lactating mothers and their breastfed infants.

## Background

Vascular endothelial growth factor (VEGF) is a multifunctional cytokine. VEGF in human breast milk, produced by lactiferous mammary gland epithelial cells, has an important function in the development and maturation of the infant digestive system [1–4]. Breast tissue itself is the target organ of VEGF as well. During pregnancy and lactation, mammary glands are markedly developed, and VEGF may play a role in mammary gland development. The concentration of VEGF in milk is significantly higher than that in serum [2]. VEGF also plays a more vital role in vasculogenesis and angiogenesis [5]. VEGF is involved in the development of choroidal neovascularization (CNV) and inflammatory retinal diseases [6]. CNV are abnormal, unmatured and leaky vessels, and their leakage can cause bleeding, intractable fibrosis and even eventual blindness [7].

Lactating woman with CNV or other retinal diseases required timely anti-VEGF treatment for better visual prognosis. Intravitreal injection of anti-VEGF drug is the most effective treatment for CNV to date [8, 9], and are the standard treatment for age-related macular degeneration (AMD), diabetic macular edema (DME) and macular edema secondary to retinal vein occlusion (RVO-ME) [10]. Contraindications of intravitreal injection of anti-VEGF drug include allergy to the drug, eye or periocular infection, and active endophthalmitis [10]. However, breastfeeding is not recommended during the treatment as a precaution [10]. Concern has been raised, but there are limited data regarding the change of VEGF concentration in human breast milk after intravitreal injection of anti-VEGF drug to women during lactation.

Previous cases report a few cases about lactating women who received intravitreal injection of anti-VEGF drug. CNV, AMD, DME and RVO-ME are the indications for receiving the intravitreal injection of anti-VEGF drug [10]. A woman who was diagnosed CNV and breastfeeding a 12-week-old infant received intravitreal injections of anti-VEGF agents, and the VEGF-A level in her breast milk was tested once a week. Initially, she had received bevacizumab, after which the milk VEGF-A level slowly decreased by 35% after 2 weeks and then recovered slowly in the following weeks. Then she changed to ranibizumab for treatment, after which the milk VEGF-A level remained stable without significant alterations [11].

A separate study reported a woman who was diagnosed CNV and breastfeeding a 1-month-old infant continuously after intravitreal injection of ranibizumab, her milk was obtained and tested for VEGF-A level once a day after treatment. The VEGF-A level in this breast milk remained mostly unchanged [12].

Another study reported that the VEGF-A level of human breast milk decreased temporarily for 3 days after intravitreal injection of ranibizumab for CNV treatment, then returned to the pre-injection level about 1 week after injection [11], which is inconsistent with the conclusion of the previously mentioned study. In summary, the first detection of VEGF-A concentration in human breast milk was carried out at least 1 day after anti-VEGF agent intravitreal injection. The changing trend of VEGF-A concentration within 24 h after intravitreal injection is still unknown.

In this study we enrolled one lactating mother with CNV of left eye. The lactating mother received four administrations of 0.5 mg intravitreal injection of ranibizumab of left eye during lactating. Breast milk was collected and tested just before injection, 1–3, 6, 12, 24, 48, and 72 h after each anti-vascular endothelial growth factor treatment. This report was the first time to detect the VEGF-A concentration in human breast milk in 1–3 h after intravitreal injection of ranibizumab, and depicted the changing trend of VEGF-A concentration in human breast milk in 72 h after injection. These data may provide more information to assess the impact of anti-VEGF treatment of lactating mothers and their breastfed infants (Table 1).

**Table 1:** Change of VEGF-A concentration in milk over time after injections

	Pre-injection (mg/L)	Post-injection (mg/L)					
		1–3 h	6 h	12 h	24 h	48 h	72 h
1st injection	8.2	7.9	6.6	7.0	10.7	12.3	9.8
2nd injection	7.2	7.1	5.1	5.5	6.9	6.4	8.5
3rd injection	N/A	9.2	8.9	9.3	16.0	15.4	12.8
4th injection	N/A	10.1	8.6	6.3	10.1	10.1	9.1

Milk samples were not collected prior to the third and fourth injections

## Case presentation

We measured the VEGF-A concentration in breast milk of a lactating patient before and after intravitreal injection of ranibizumab (Lucentis, Novartis). In June 2018, a 30-year-old woman breastfeeding a 6-month-old baby was diagnosed as idiopathic CNV of left eye in Eye Hospital of Wenzhou Medical University. She was physically healthy without hypertension, diabetes mellitus and other noteworthy medical history. She received four administrations of 0.5 mg ranibizumab intravitreal injection of the left eye. Blood pressure, heart rate, respiration, pulse and temperature were measured before and after each injection. The mother also received a detailed slit lamp examination, intraocular pressure examination and fundus examination, before and after each injection to avoid adverse effects. The patient received a physical and ophthalmic reexamination in a week, a month and 3 months after each injection. No adverse effects or side effects were reported during the treatment period. The patient stopped feeding her breast milk to her baby for 3 days after anti-VEGF treatment. In order to maintain lactation, she expressed her milk. The milk samples obtained just before injection, 1–3, 6, 12, 24, 48, and 72 h after each anti-VEGF treatment were tested for the milk VEGF-A concentration. Milk samples were centrifuged, and the fat portion was removed. Milk VEGF-A concentration was measured with an immunoassay analyser (R&D DVE00 Human VEGF Quantikine ELISA Kit). We obtained written informed consent from the patient, and this case study is in accordance with the tenets of the Declaration of Helsinki.

**Fig. 1:**
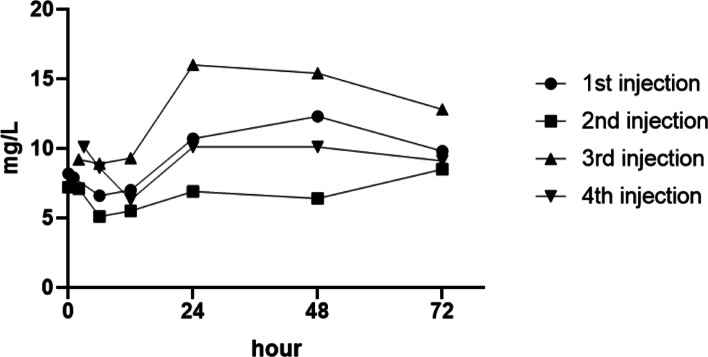
Change of VEGF-A concentration in milk over time after injections

Milk samples were not collected prior to the third and fourth injections. The change in VEGF-A concentration in breast milk showed the same trend after each injection (Fig. 1), decreasing significantly within 6–12 h (the maximum decrease was less than 40%), and increased to pre-injection levels by 24 h after injection.

## Discussion

Intravitreally injected anti-VEGF agents can reach the breast milk by circulation. Elevated levels of anti-VEGF agents via the bloodstream and breast milk result in a reduction of VEGF levels in breast milk. Ranibizumab is a 48 kDa recombinant humanized monoclonal antibody fragment against VEGF-A; it lacks a fragment crystallizable (Fc) region and is rapidly cleared from the bloodstream [13, 14]. Avery et al. reported that the reduction in plasma VEGF level was greater with intravitreal injection of aflibercept, and less with ranibizumab [15]. Agostini et al. reported that the plasma VEGF level decreased significantly, and the milk VEGF level decreased by 30% about 8 weeks after intravitreal injection of bevacizumab, while the plasma and milk VEGF levels returned to the pre-injection level after only 1 week after intravitreal injection of ranibizumab [11]. These studies indicated that the plasma and milk VEGF levels were less affected by treatment of ranibizumab than by aflibercept or bevacizumab. Therefore, ranibizumab was chosen for treatment of the lactating patient in this study.

Another study reported that continuous milk production was crucial for maintaining breast milk VEGF-A level. Two 37-year-old lactation female patients diagnosed with myopic choroidal neovascularization received ranibizumab treatment. One was breastfeeding continuously, the VEGF-A level in this patient’s breast milk remained mostly unchanged throughout all study time points (1 h before injection, days 1 through 7, and days 14, 21, and 28 after injection). The other patient stopped breastfeeding; the VEGF-A level in this patient’s breast milk was reduced nearly 50% from baseline to day 1, and down to 20% of baseline on day 28 after treatment [11]. Since breast milk production reduced, the concentration of VEGF itself dropped, and is not entirely a result of intravitreal injection of anti-VEGF agents. The reduction of VEGF-A level in breast milk was small and transient in the patient who received intravitreal injection of ranibizumab in our study, which may be related to the regular milk expression, to ensure continued production after the treatment. The concentration of VEGF-A in human breast milk changed with time after the intravitreal injection of ranibizumab. In this study, we found that the human breast milk VEGF-A level, in a patient continuing to lactate decreased within a few hours after intravitreal injection of ranibizumab, returning to a normal level after about 1 day.

A study reported that ranibizumab was detected in milk starting on day three, with generally increasing levels over time after intravitreal injection of ranibizumab in a patient who stopped breastfeeding, while concentration of ranibizumab levels in breast milk remained at a low level if a person continued breastfeeding [11]. Two lactating women who received injections were tested for bevacizumab level in their breast milk, and the milk samples, collected 1.5 and 7 h after intravitreal injection of bevacizumab, were negative for bevacizumab [16]. The concentration of anti-VEGF drug in human breast milk may provide an indication for breastfeeding in women who receive intravitreal injection of anti-VEGF drug. Anti-VEGF drug will enter into the infants’ digestive system in milk by breastfeeding, reduce the original VEGF level in infants’ digestive system, and then affect the development and maturation of infants’ digestive system.

### Limitation

The concentration of ranibizumab in breast milk is very important information to evaluate the impact of anti-VEGF agents intravitreal injection to lactating mothers and their breastfed infants. However, the concentration of ranibizumab in milk was not measured in this study. This was one limitation of this study. Another limitation was the small sample size, because such a patient was relatively rare.

## Conclusions

This case report depicted the changing levels of VEGF-A concentration in human breast milk in 72 h after intravitreal injection of ranibizumab. VEGF-A in human breast milk, which was produced continually, decreased little during the few hours after intravitreal injection of ranibizumab, and returned to a normal level in about 1 day. These data may provide more information to analyze the impact of anti-VEGF agent intravitreal injection of lactating mothers and their breastfed infants.

## Data Availability

All data generated or analysed during this study are included in this published article.

## Abbreviations

AMD: Age-related macular degeneration
CNV: Choroidal neovascularization
DME: Diabetic macular edema
ME: Macular edema
RVO: Retinal vein occlusion
VEGF: Vascular endothelial growth factor
